# *GAS2* encodes a 2-oxoglutarate dependent dioxygenase involved in ABA catabolism

**DOI:** 10.1038/s41467-023-43187-1

**Published:** 2023-11-22

**Authors:** Theo Lange, Nadiem Atiq, Maria João Pimenta Lange

**Affiliations:** 1 Technische Universität Braunschweig, Braunschweig, Germany; 2 Present Address: Julius Kühn Institute (JKI), Institute for Plant Protection in Horticulture and Urban Green, Braunschweig, Germany

**Keywords:** Plant hormones, Enzymes, Gibberellins

**arising from** H. Liu et al. *Nature Communications* 10.1038/s41467-019-09467-5 (2019)

Liu et al.^1^ recently reported the characterisation of *Arabidopsis thaliana* GAS2 (Gain of Function in ABA-modulated Seed Germination 2), which was described as an enzyme catalysing the stereospecific hydration of GA_12_ to produce GA_12_ 16, 17-dihydro-16α-ol (DHGA_12_). A second paper describes the conversion of GA_12_ to an unidentified product by GAS2 and reports that this enzyme does not convert ABA^2^. As previously reported^3^, we did not find any conversion of [17-^14^C]-labelled or [1-,7-,12-,18-^14^C_4_]-labelled GA_12_ by GAS2. Instead, we present here data showing that the recombinant GAS2 enzyme catabolizes abscisic acid (ABA) to phaseic acid (PA) and further to a second product, putative 8’-carboxy-ABA (compound A; Fig. 1a).

**Fig. 1:**
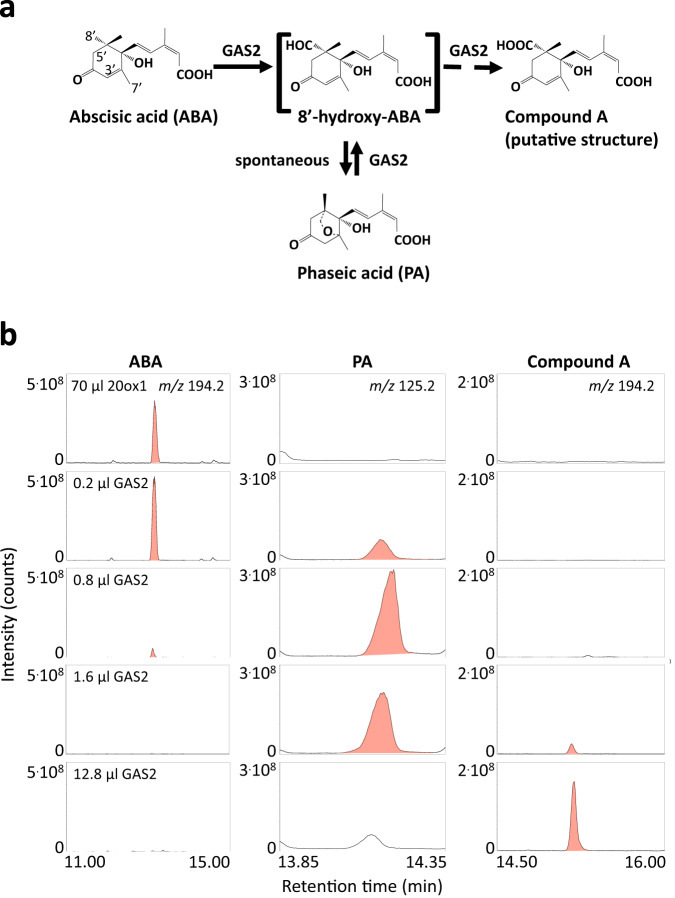
GAS2 is an ABA catabolising oxidase. **a** The proposed ABA catabolic pathway catalysed by GAS2. **b** Metabolism of 3’,5’,5’,7’,7’,7’-*d*_*6*_-ABA incubated with 70 µL cell lysate containing recombinant ATGA20ox1 and cofactors (negative control, top lane). Metabolism of *d*_*6*_-ABA incubated with indicated volumes of cell lysates containing recombinant GAS2 and cofactors as described in Methods. Chromatograms of characteristic single ions are shown in the first row for ABA (extracted ion *m/z* 194.2), in the middle row for PA (*m/z* 125.2), and in the right row for compound A (*m/z* 194.2). Compounds that were identified on the basis of their full scan mass spectra of the methyl ester derivatives are shown in red. Similar results were obtained with two recombinant GAS2 preparations, produced from independent isolated *E.coli* clones, each were incubated at seven different lysate concentrations.

ABA is known to be oxidatively catabolized by cytochrome P450 monooxygenases^4^. Three different hydroxylation pathways have been described that oxidise one of the methyl groups of the ring structure, at C-7’, C-8’, or C-9’. 8’-hydroxylation appears to be the major pathway, and Arabidopsis *CYP707As* encode ABA 8’-hydroxylases that initiate the oxidation at C-8’ to produce 8’-hydroxy-ABA, which spontaneously autoisomerises to PA^5,6^. This final cyclization step is reversible, and the final equilibrium is at PA^7^ (Fig. 1a).

For this study, Arabidopsis GAS2 was heterologously expressed in *E. coli* according to our standard protocols^8,9^. Surprisingly, recombinant GAS2 does not convert 17,17-d2-GA_12_ under either our incubation conditions or those described by Liu et al. ^1^ and by Xiong et al.^2^ (Supplementary Fig. 1, right column). Note that Liu et al. show in Figure 3^1^ that 17,17-*d2*-DHGA_12_ accumulated after 5, 30, and 120 min, although they state in the same paper that “fresh cofactors were added after 2, 4, 6 and 8 h” and that the “reaction mixtures were incubated overnight at 30 °C”. In contrast, recombinant GAS2 efficiently converts deuterated ABA regardless of the incubation conditions, as long as cofactors essential for the activity of 2-oxoglutarate-dependent dioxygenases are added (Supplementary Fig. 1, left column). As a control, Arabidopsis gibberellin 20-oxidase 1 (AtGA20ox1) was expressed in *E. coli* in the same way. No products are formed with AtGA20ox1 and deuterated ABA (Fig. 1b, top lane), indicating that the *E. coli* cell lysate is devoid of ABA-oxidising activities. Under our incubation conditions, lysate volumes of 1.6 µL or more are sufficient to completely convert the ABA substrate (Fig. 1b, left row). At low lysate volumes, an intermediate product is formed first, which disappears at high lysate volumes (PA, Fig. 1b, middle column) when a second product appears (compound A, Fig. 1b, right column).

Mass spectra and KRI of the intermediate product from incubations of GAS2 with ABA correspond to PA (Table 1). Interestingly, when 3′,5′,5′,7′,7′,7′-*d6*-ABA is used as substrate, *d3*-PA is formed while compound A retains its *d6*-labels. This indicates an exchange of deuterium for hydrogen, which preferentially occurs during the methylation of PA by diazomethane^10^. The two deuterium atoms at the 5′-carbon are the most easily exchanged. In addition, the deuterium atom at the 3′-carbon of PA is abstracted by diazomethane. When 7′,7′,7′-*d3*-labelled PA is the substrate it retains the *d3*-labels, indicating that the three deuterium atoms at the 7′-carbon are unlikely to be exchanged (Table 1). The different stability of the deuterium atoms in ABA and PA can be explained by the structural differences between the two compounds^10^ and could indicate that compound A is structurally closer related to ABA than to PA.

**Table 1:** Metabolism of *d6*-ABA, *d3*-PA, ABA, and PA by cell lysates from *E. coli* transformed with AtGAS2 in pET21a

Cell lysate (µL)	Substrates	Compounds formed	KRI	Characteristic ions at *m/z* (% relative of base peak)^a^
	ABA		2086	278[M^+^](0), 260(2), 246(2), 222(2), 205(6), 190(100), 162(51), 147(12) 134(58), 125(40), 112(13)
0.8		PA	2144	294[M^+^](3), 276(5), 262(3), 244(7), 217(10), 204(6), 194(4), 177(16), 167(23), 154(21), 139(37), 125(100), 122(66), 109(27), 94(44), 83(17)
3.2		Cmp A	2202	322[M^+^](1), 290(5), 272(5), 249(6), 231(10), 213(6), 203(7), 190(100), 175(9), 162(42), 134(71), 125(82), 112(9), 101(10)
	PA		2146	294[M^+^](4), 276(6), 262(3), 244(8), 217(10), 204(6), 194(4), 177(18), 167(27), 154(24), 139(42), 125(100), 122(71), 109(28), 94(42), 83(14)
70		Cmp A	2201	322[M^+^](1), 290(4), 272(5), 249(7), 231(10), 213(6), 203(7), 190(100), 175(9), 162(44), 134(71), 125(84), 112(9), 101(09)
	*d6*-ABA^b^		2083	284[M^+^](0), 266(2), 252(2), 226(2), 211(4), 194(100), 166(55), 151(7), 138(58), 125(43), 112(13)
0.8		*d3*-PA	2144	297[M^+^](6), 279(10), 265(5), 247(13), 220(17), 204(11), 194(7), 177(27), 170(27), 154(36), 142(50), 125(75), 122(100), 112(11), 94(58), 83(17)
12.8		*d6*-Cmp A	2195	328[M^+^](1), 296(5), 278(4), 255(5), 237(6), 219(3), 209(5), 194(88), 181(6), 166(42), 138(72), 125(100), 112(13), 103(10)
	*d3*-PA^c^		2143	297[M^+^](4), 279(8), 265(5), 247(11), 220(14), 204(11), 194(7), 177(25), 170(23), 154(34), 142(43), 125(71), 122(100), 112(11), 94(64), 83(25)
70		*d3*-Cmp A	2199	325[M^+^](1), 293(5), 275(6), 252(7), 234(10), 216(7), 206(10), 193(88), 178(9), 165(45), 137(79), 125(100), 112(15), 101(10)

Identification of PA and *d3*-PA by GC-MS on the basis of authentic reference compounds, Kovats retention indices (KRI) of the methyl ester derivatives, and reference mass spectra^14^. Added cell lysate volumes are as indicated. Similar results were obtained in incubations with recombinant GAS2, produced from *n* independent isolated *E.coli* clones and *d6*-ABA (*n* = 7), *d3*-PA (*n* = 2), and PA (*n* = 2).

^a^Based on ions above a mass/charge ratio (*m/z*) of 60.

^b^*d6*-ABA was (3′,5′,5′,7′,7′,7′-*d6*)-labelled and ^c^*d3*-PA was (7′,7′,7′-*d3*)-labelled.

There is no known ABA catabolite that matches the mass spectrum of compound A. In fact, compound A shares several fragment ions with ABA^11^, again suggesting structural similarities between the two compounds (Table 1). Unlabelled compound A shows a molecular ion (M^+^) of 322 mass units in good agreement with ABA, containing an additional carboxyl group (as a methyl ester) most likely at the 8′-position. This structure suggests that GAS2 catalyses the conversion of ABA and PA to 8′-carboxy-ABA via 8′-hydroxy-ABA, and 8′-aldehyde-ABA (the structure for the latter is not shown in Fig. 1a). Such intermediates have not been isolated, possibly because of their instability or rapid conversion^12^. However, such a stepwise oxidation pathway from methyl to the carboxyl group via the alcohol and aldehyde has been found in reactions catalysed by other 2-oxoglutarate-dependent dioxygenases, including gibberellin 20-oxidases^3^. In addition, compound A, together with ABA and PA, was identified as an endogenous compound in 31-day-old Arabidopsis shoots that had been drought-stressed for 17 days and then rehydrated for 3 days (Supplementary Table 1a). No endogenous DHGA_12_ was detected in this material (Supplementary Table 1b). Liu et al. ^1^ identified endogenous DHGA_12_ in imbibed Arabidopsis seeds, but our attempt to reproduce this finding was unsuccessful (Supplementary Fig. 2), suggesting that DHGA_12_ may not consistently accumulate to appreciable levels (at least not in our conditions) arguing against a substantial biological role.

Xiong et al.^2^ observed that Arabidopsis *GAS2* overexpressing lines (here called GIM2) have decreased endogenous ABA levels, whereas the Arabidopsis knock-out mutant has increased endogenous ABA levels. Furthermore, *GAS2* overexpressing lines show reduced sensitivity to ABA in germination and early seedling development compared to WT and increased sensitivity to ABA in *GAS2* loss-of-function mutants^1^. In agreement with these observations and our own results, we conclude that the main function of the 2-oxoglutarate-dependent dioxygenase GAS2 is to initiate ABA catabolism, and that gibberellin anabolism, as proposed by Liu et al.^1^, maybe only a side reaction of this enzyme.

## Methods

### Heterologous expression of recombinant GAS2 and GA20ox1

*AtGAS2* (AT2G36690) and *AtGA20ox1* (AT4G25420) coding sequences were custom synthesised in the pET21a expression vector (BioCat), and sequence identities were verified by sequencing on both strands. The respective plasmid DNAs were used to transform BL21 Star^TM^ *E. coli* (Invitrogen) according to the manufacturer’s instructions. From the transformation reaction, 200 µL were used to inoculate 10 mL L-broth medium supplemented with carbenicillin (50 µg·mL^−1^), and transformed *E. coli* were grown for 16 h at 37 °C with shaking (250 rpm). From the previous *E. coli* culture, 2 mL were grown shaking (250 rpm) at 30 °C in 100 mL 2× YT broth with carbenicillin to OD_600_ between 0.7 and 1. Expression was induced by the addition of IPTG to 1 mM; the culture was grown for a further 2 h at 30 °C and harvested by centrifugation (5,000 × *g* for 10 min at 4 °C). The pellet was resuspended in 200 µL of 200 mM Tris, pH 7.8 at 4 °C, containing 10 mM DTT and 1 mg^.^mL^−1^ lysozyme and the cells were disrupted by incubating on ice for 30 min, followed by four cycles of freezing in liquid N_2_ and thawing. DNaseI (20U in 10 µL) were added to the *E. coli* cell lysate and incubated for 15 min at 20 °C. The lysates were centrifuged at 30,000 × *g* for 30 min at 4 °C to give approximately 500 µL of supernatant, which was used for enzyme assays^8,9^.

### Enzyme assays and purification of incubation products

3′,5′,5′,7′,7′,7′-*d*_*6*_-labelled ABA was purchased from OlChemIm, Czech Republic. PA and 7′,7′,7′-*d3*-PA were gifts from Professor Eiji Nambara (University of Toronto, Canada). Preparations of *E. coli* cell lysates were incubated in a total volume of 100 µL containing 100 mM Tris-HCl, pH 7.0 at 30 °C for 16 h with 2-oxoglutarate and ascorbate (100 mM each, final concentrations), FeSO_4_ (0.5 mM), catalase (1 mg·mL^−1^), and the substrates (5 µL in methanol) ABA (500 ng), 3′,5′,5′,7′,7′,7′-*d*_*6*_-labelled ABA (500 ng), PA (500 ng), or 7′,7′,7′-*d3*-labelled PA (50 ng). Variations to the standard incubation conditions are indicated in the individual experiments. The enzymatic reactions were stopped by adding 10 µL acetic acid, which lowered the pH to about 3.2 and the incubation products were extracted two times with 500 µL methanol.

The methanolic extracts were diluted with water (10 mL), containing 1% (v/v) acetic acid. The samples were loaded onto a C_18_ reverse-phase Sep-Pak cartridge (Waters) and the cartridge was washed with water (2 × 10 mL), containing 1% (v/v) acetic acid. ABA incubation products were eluted from the cartridge with 80% (v/v) methanol-water, containing 1% (v/v) acetic acid. The eluates were dried under N_2_ and redissolved in 200 µL methanol-water, containing 1% acetic acid (1:1), and applied to a C_18_ reverse-phase column (10 cm long, 8 mm i.d., 4 mm particle size, Nova-Pak liquid chromatography cartridge in a RCM100 radial compression system; Waters), which was eluted using gradients of increasing methanol in water, containing 1% acetic acid, at 1 mL^.^min^−1^ as follows: 50% methanol, followed by five 0.5-min steps to 57.4%, 60.6%, 61.8%, 62.3%, 62.5%, one 5-min step to 62.7%, one 1-min step to 63.2% and seven 2-min steps to 64.3%, 67.2%, 70.5%, 74.9%, 81%, 89% and 100% methanol^13^. PA and compound A eluted between 3 and 6 min, and ABA between 6 and 9 min. The dried HPLC-fractions were redissolved in 100 µL methanol and methylated with 100 µL ethereal diazomethane. Samples were then transferred to glass ampoules, dried, and redissolved in 2–4 µL dichloromethane and subjected to full-scan GC-MS analysis (see below).

### GC-MS analysis

The derivatized samples were analysed using a Thermo Scientific MS system ISQ 7000 with Advanced Electron Ionization (AEI) source equipped with a Thermo Scientific Trace 1300 gas chromatograph (GC-MS). The performance of the GC-MS was evaluated every day prior analyses by injecting authentic GA_4_ as Me ester TMSi ether (0.1 ng in 1 µL in MSTFA). Samples (1–2 µL) were injected into an Agilent DB-5MS UI capillary column (30 m long, 0.25 mm i.d., 0.25 µm film thickness; Agilent, USA) at an oven temperature of 60 °C. The split value (30:1) was opened after 1 min, after which the temperature was increased by 45 °C min^−1^ to 175 °C and then with 4 °C min^−1^ to 280 °C. The He inlet was pneumatic pressure controlled at a constant flow rate of 1.2 mL^.^min^−1^, and the injector, transfer line, and source temperatures were 280 °C, 280 °C and 250 °C, respectively. Mass spectra were acquired from 5 to 30 min after injection at an electron energy of 45 eV from 60 to 660 atomic mass units at 0.2 s per scan. For determination of Kovats retention index (KRI), 0.5 µL solution of 1 cm^2^ parafilm in 2 mL hexane was injected.

### Reporting summary

Further information on research design is available in the Nature Portfolio Reporting Summary linked to this article.

### Supplementary information

Supplementary Information

Reporting Summary

## Data Availability

The mass spectrometry data have been deposited to the Metabolomics Workbench with the Study ID ST002810 [10.21228/M8M13Z].
