# Supplementary material for: GAS2 encodes a 2-oxoglutarate dependent dioxygenase involved in ABA catabolism
Source: Nat Commun. 2023 Nov 22;14:7602. doi: 10.1038/s41467-023-43187-1 (PMC10663614; doi:10.1038/s41467-023-43187-1)
Supplement: Supplementary file 1 — Supplementary Information [file 41467_2023_43187_MOESM1_ESM.docx]

***GAS2* encodes a 2-oxoglutarate dependent dioxygenase involved in ABA catabolism**

Lange *et al*.

**Supplementary Methods**

**Enzyme assays with GA_12_ as substrate and extraction of incubation products.**17,17-*d2*-labelled GA_12_ were purchased from OlChemIm, Czech Republic. Enzyme assays were performed as described in Methods for ABA enzyme assays using 17,17-*d2*-labelled GA_12_ (5 ng in 2 µL methanol) as the substrate. Variations to the standard incubation conditions are indicated in the individual experiments. The enzymatic reactions were stopped by adding 10 µL acetic acid, which lowered the pH to about 3.2. The incubation products were extracted by methanol (two times with 500 µL methanol) and dried under N_2_. The extracts were redissolved in methanol (2 mL) and after loading onto an anion-exchange column (BondElut DEA, Varian), the column was washed with methanol (6 mL). The gibberellins eluted with methanol, containing 1% (v/v) acetic acid (6 mL). The eluates were dried under N_2_ and redissolved in methanol (1 mL). The samples were further purified by solid phase extraction on C_18_ reverse-phase Sep-Pak cartridges as described in Methods for purification of ABA incubation products. The eluates were dried under N_2_, redissolved in 100 µL methanol and methylated with 100 µL ethereal diazomethane. Samples were then transferred to glass ampoules, dried and trimethylsilylated with 2-4 µL *N*-methyl-*N*-trimethylsilyltrifluor-acetamide (MSTFA; Macherey and Nagel) at 80°C for 30 min and subjected to full-scan GC-MS analysis (see Methods).

**Purification and analysis of endogenous ABA derivatives and gibberellins.**Three biological replicates were harvested from 31-day-old shoots of Arabidopsis Col-0 plants, which were not watered for 17 days from day 11 and then rehydrated for 3 days. Plant materials were freeze dried and pulverized. For analysis of endogenous ABA and its derivatives, material from the three biological replicates described above were pooled (0.03 g from each). For extraction of endogenous compounds, 80% methanol-water (3 mL) was added to the Arabidopsis plant material and the extract was stirred for 30 min at 4°C. After centrifugation at 4000x*g*, the pellet was reextracted with methanol (3 mL) for 30 min and recentrifuged. The reextraction procedure was repeated two times. The combined methanol extracts were evaporated to dryness, resuspended in water (6 mL), and adjusted to pH 8.0 (1 M KOH). Solvent partition was performed using ethyl acetate (four times, 3 mL). The aqueous phase was adjusted to pH 3 (acetic acid) followed by solvent partition with ethyl acetate (four times, 3 mL). The combined ethyl acetate fractions were dried under N_2_ and redissolved in methanol (1 mL). The extracts were purified via C_18_ reverse-phase Sep-Pak cartridge and C_18_ reverse-phase HPLC as described in Methods for purification of ABA incubation products. After HPLC, ABA and its derivatives eluted between 5 and 10 min. Samples were then transferred to glass ampoules, dried, and redissolved in 2-4 µL dichloromethane and subjected to full-scan GC-MS analysis (see Methods).

For quantitative analysis of endogenous GAs from the dehydrated and rehydrated Arabidopsis Col-0 plants described above, each of the three biological replicates (0.02 g) were spiked with 17,17-*d2*-GA_12_ (2.5 ng) and 17,17-*d2*-DHGA_12_ (0.4 ng). After HPLC, authentic 17,17-*d2*-DHGA_12_ eluted between 21-24 mL, which was prepared from respective labelled GA_12_ as described by Liu et al.^1^ for the chemical synthesis of DHGA_12_. The identity of the chemically prepared DHGA_12_ was verified in enzyme assays as described above for GAS2 using recombinant AtGA20ox1 and a mixture of recombinant AtGA20ox1 and AtGA3ox1 (1:1, v/v), resulting in the known DHGAs products^14^, GA_10_ and GA_2_, respectively, whose identity were confirmed by full-scan GC-MS. KRI of 17,17-*d2*-DHGA_12_ as Me ester TMSi ether is 2597, with MS of m/z (rel. int.): 452[M^+^](4), 437(10), 405(18), 393(12), 363(10), 330(31), 303(17), 289(100), 288(89), 243(48), 212(29), 201(43), 181(24), 159(15), 145(17), 132(27), 119(23), 107(34). Endogenous GAs were extracted by solvent partition as described above. The combined ethyl acetate fractions were dried under N_2_, redissolved in methanol (2 mL) and further purified by extraction on DEA anion exchange cartridges as described above for extraction of gibberellin enzyme assays. The samples were then further purified using C_18_ reverse-phase Sep-Pak cartridge as described in Methods for purification of ABA incubation products. The samples were methylated and trimethylsilylated as described above and analysed by GC-MS in the selected ion monitoring mode (SIM, for settings see Methods). The ions monitored for quantification were 288 and 286, and 330 and 328 for DHGA_12_ and 330 and 328, and 302 and 300 for GA_12_. Identiﬁcation was conﬁrmed on the basis of retention time and the co-occurrence of additional ions. Endogenous levels were calculated on the basis of peak areas, after corrections were made for the contribution of naturally occurring isotopes and for the presence of unlabelled GAs in the internal standards, when necessary^15^.

For analysis of endogenous gibberellins from 0.6 g dry Arabidopsis Col-0 seeds imbibed in water at 4°C for 20 h, the plant material was pulverized under liquid nitrogen. Gibberellins were extracted by solvent partition, solid phase extraction on DEA anion exchange and C_18_ reverse-phase Sep-Pak cartridges as described above, and then separated by C_18_ reverse-phase HPLC as described in Methods for purification of ABA incubation products. R/T of authentic 17-^14^C-labeled DHGA_12_ by HPLC was 23.13 min. The samples were methylated and trimethylsilylated as described above and analysed by full-scan GC-MS (see Methods).

**Supplementary References**

15. Lange, T. et al. The class III gibberellin 2-oxidases AtGA2ox9 and AtGA2ox10 contribute to cold stress tolerance and fertility. *Plant Physiol.* **184**, 478-486 (2020).


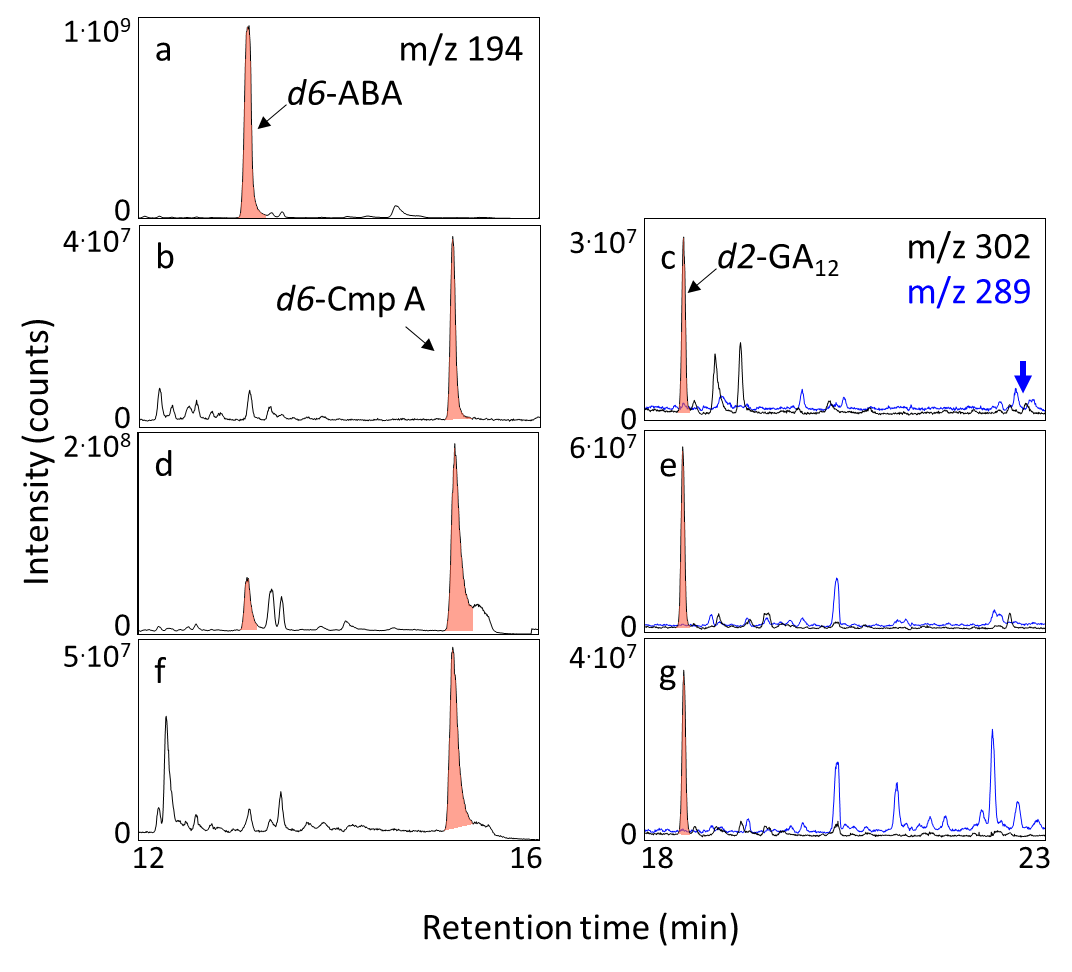


**Supplementary Fig. 1** **| Metabolism of ABA and GA_12_ by GAS2.**

**a-c** Incubations with GAS2 cell lysate (10 uL) and 3’,5’,5’,7’,7’,7’-*d6*-ABA (50 ng, **a,b**) or 17,17-*d2*-GA_12_ (5 ng, **c**) without (**a**) or with cofactors (**b,c**) for 3 h as described in Methods. **d,e** Incubations as described by Liu et al.^1^; GAS2 cell lysate (20 µL) with 3’,5’,5’,7’,7’,7’-*d6*-ABA (100 ng, **d**) or 17,17-*d2*-GA_12_ (10 ng, **e**) and (final concentrations) 14.3 mM ascorbate, 14.3 mM 2-oxoglutarate, 1.8 mM FeSO_4_, and 3.5 mg/mL catalase in 50 mM Tris/HCl, pH 7.8 in a total volume of 224 µL, for 2 h at 30°C. **f,g** Incubations according to Xiong et al.^2^ with 3’,5’,5’,7’,7’,7’-*d6*-ABA (50 ng, **f**) or 17,17-*d2*-GA_12_ (5 ng, **g**) and (final concentrations) 5 mM ascorbate, 4 mM 2-oxoglutarate, and 0.5 mM FeSO_4_ in 100 mM Tris/HCl, pH 7.5 in a total volume of 100 µL, for 3 h at 30°C. **a,b,d,f** The ABA incubation products were extracted and purified as described in Methods. The RP-HPLC fractions, which eluted between 5 and 10 min, contained ABA and compound A, and were further analysed by GC-MS as their methyl esters (shown as extracted single ion chromatograms at m/z 194). **c,e,g** The GA_12_ incubation products were extracted and purified as described for the endogenous GAs and analysed by GC-MS as their methyl esters trimethylsilyl ethers (shown as extracted single ion chromatograms for *d2*-GA_12_ at m/z 302 (black lane) and for *d2*-DHGA_12_ at m/z 289 (blue lane)). Compounds identified on the basis of their full scan mass spectra are shown in red. The blue arrow indicates the position in the gas chromatogram where authentic *d2*-DHGA_12_ elutes.


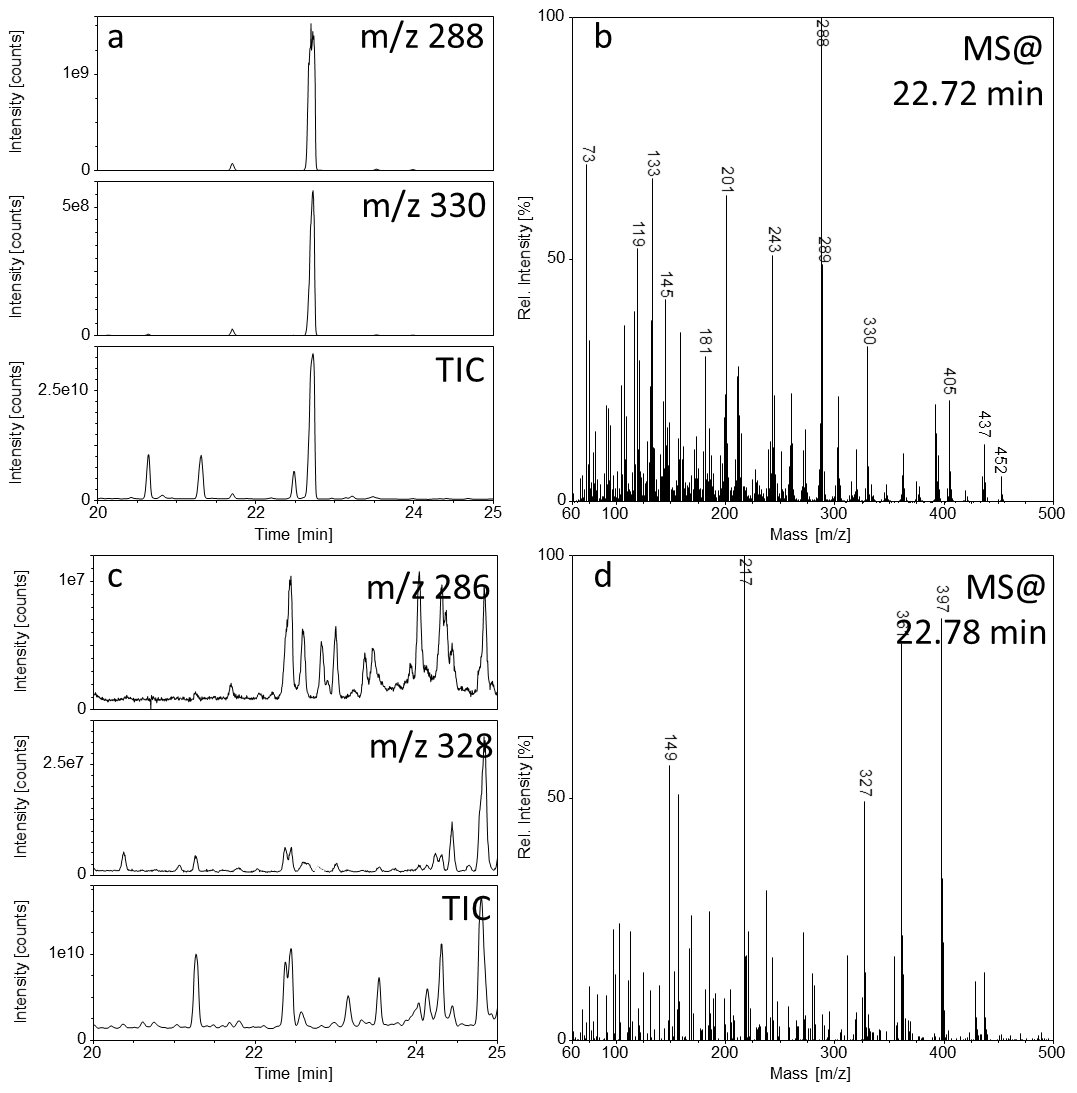


**Supplementary Fig. 2 | Endogenous compounds from imbibed Arabidopsis Col-0 seeds.**

**a,b** Authentic 17-^14^C-labelled DHGA_12_. **a** From top to bottom: Single ion chromatograms at m/z 288 and m/z 330 and total ion chromatogram (TIC) of authentic 17-^14^C-labelled DHGA_12_ (RP-HPLC, R/T = 23.13 min). **b** Mass spectrum of authentic 17-^14^C-labelled DHGA_12_. **c,d** Endogenous compounds extracted from 0.6 g dry Arabidopsis Col-0 seeds (n=1) imbibed in water at 4°C for 20 h. **c** From top to bottom: Single ion chromatograms at *m/z* 286 and *m/z* 328 and TIC of compounds eluted in RP-HPLC fraction 21-24 ml. **d** The mass spectrum recorded at the expected GC R/T of DHGA_12_. No endogenous DHGA_12_ was detected.

**Supplementary Table 1** **| Endogenous ABA, PA, compound A, and GAs in shoots of 31 day-old rehydrated Arabidopsis Col-0.**

Arabidopsis Col-0 was grown under our standard conditions^15^. From day 11 on, watering was stopped for 17 days, and then plants were rehydrated and harvested three days later. **a** GC-MS identification of endogenous ABA, PA, and compound A based of mass spectra and Kovats retention indices (KRI) of the methyl ester derivatives (n = 2). **b** Endogenous GA_12_ and DHGA_12_ levels (means ± SD) of three biological replicates (in ng^.^g^-1^ dry weight). Endogenous DHGA_12_ was not detected (marked n.d.), but internal standard was recovered. Similar results were obtained with one biological replicate (*n*=2).

**a** KRI Characteristic ions at *m/z* (% relative intensity of base peak)^a^

ABA 2096 278[M^+^](1), 260(3), 246(4), 222(4), 205(12), 190(100), 162(56), 147(17), 134(52), 125(35), 112(5)

PA 2150 294[M^+^](2), 276(4), 262(8), 244(6), 217(13), 204(3), 177(7), 154(17), 139(39), 125(100), 122(68), 109(24), 94(28)

Cmp A 2205 322[M^+^](1), 290(5), 272(6), 249(5), 231(12), 213(7), 203(6), 190(100), 175(17), 162(36), 134(64), 125(82), 112(12), 101(10)

^a^Based on ions above a mass-to-charge ratio (*m/z*) of 60.

**b** GA_12_ DHGA_12_

7.8 ±2.9 n.d.
